# Developing a Consensus Statement to Target Oral Health Inequalities in People With Severe Mental Illness

**DOI:** 10.1111/hex.14163

**Published:** 2024-08-03

**Authors:** Masuma Pervin Mishu, Vishal Aggarwal, David Shiers, Emily Peckham, Gordon Johnston, Easter Joury, Carolyn A. Chew‐Graham, Katie Goodall, Emma Elliott, Paul French, Rebecca Harris, Louise Laverty, Jasper Palmier‐Claus

**Affiliations:** ^1^ Institute of Epidemiology and Health Care University College London London UK; ^2^ School of Dentistry University of Leeds Leeds UK; ^3^ Psychosis Research Unit Greater Manchester Mental Health NHS Trust Manchester UK; ^4^ University of Manchester Manchester UK; ^5^ School of Medicine Keele University Keele UK; ^6^ School of Health Sciences Bangor University Bangor UK; ^7^ Independent Peer Researcher; ^8^ Institute of Dentistry Queen Mary University of London London UK; ^9^ School of Nursing & Public Health Manchester Metropolitan University Manchester UK; ^10^ Leeds Teaching Hospitals Trust Leeds UK; ^11^ Manchester Metropolitan University, Pennine Care NHS Manchester UK; ^12^ University of Liverpool Liverpool UK; ^13^ Royal Liverpool University NHS Foundation Trust, Department of Public Health, Policy and Systems, Institute of Population Health University of Liverpool Liverpool UK; ^14^ NIHR Applied Research Collaboration Greater Manchester (NIHR ARC GM)—Digital Health, Centre for Health Informatics University of Manchester Manchester UK; ^15^ Spectrum Centre for Mental Health Research Lancaster University Lancaster UK; ^16^ Lancashire & South Cumbria NHS Foundation Trust Lancashire UK

**Keywords:** consensus statement, mental illness, oral health

## Abstract

**Introduction:**

Oral diseases are more prevalent in people with severe mental illness (SMI) compared to those without mental illnees. A greater focus on oral health is needed to reverse unacceptable but often neglected oral health inequality in people with SMI. This provided the impetus for developing ‘The Right to Smile’ consensus statement. We aimed to develop and disseminate a consensus statement to address oral health inequality, highlighting the main areas for concern and recommending an evidence‐based 5‐year action plan to improve oral health in people with SMI.

**Methods:**

The Right to Smile consensus statement was developed by experts from several professional disciplines and practice settings (mental, dental and public health) and people with lived experience, including carers. Stakeholders participated in a series of online workshops to develop a rights‐based consensus statement. Subsequent dissemination activities were conducted to maximise its reach and impact.

**Results:**

The consensus statement was developed to focus on how oral health inequalities could be addressed through a set of 5‐year improvement targets for practice, policy and training. The consensus was reached on three 5‐year action plans: ‘Any assessment of physical health in people experiencing SMI must include consideration of oral health’, ‘Access to dental services for people with SMI needs to improve’ and ‘The importance of oral health for people experiencing SMI should be recognised in healthcare training, systems, and structures’.

**Conclusion:**

This consensus statement urges researchers, services and policymakers to embrace a 5‐year action plan to improve oral health for people with SMI.

**Patient or Public Contribution:**

The team included people with lived experience of SMI, their carers/family members and mental and dental health service providers. They were involved in every stage of developing the consensus statement, from conception to development and dissemination.

## 1 Introduction

People experiencing severe mental illness (SMI) face significant problems with their oral health, including high rates of tooth decay, tooth loss and periodontal (gum) disease [1]. A recent umbrella review indicated that people with SMI are nearly three times more likely to lose all of their natural teeth and five times more likely to have tooth decay compared to the general population [2]. Poor oral health can have a profound impact on people's general health and quality of life [3]. It can affect basic functions such as eating and speaking, a person's confidence and self‐esteem and social interactions [4]. Oral diseases are also related to other systemic diseases such as diabetes and cardiovascular disease [5, 6]. Therefore, improving the oral health of people with SMI is of paramount importance.

The causes of oral diseases in people with SMI are likely multifactorial and may include difficulties with self‐care [7], high dietary intake of sugar, cigarette smoking, tobacco and alcohol consumption [8] and side effects from psychotropic medications (e.g., dry mouth) [9]. People with mental health difficulties are less likely to access dental services for routine dental check‐ups and treatment [7], potentially driven by barriers related to their mental ill health [10] and a lack of social support systems for accessing dental care [11]. There are also dental practice and health system factors that create barriers to dental care for patients with SMI [12]. In the United Kingdom, this health inequality is exacerbated by deteriorating access to NHS dentists following the COVID‐19 pandemic, which disrupted services and speeded up the shift of NHS dental practices to the private sector [13]. As the gap between demand and supply has grown, particular subgroups, such as people with SMI, have found it difficult to access care, especially if they are new patients to the practice or have a lot of treatment needs [14].

Dental diseases can be prevented with good oral hygiene and dietary habits, early diagnosis and proactive rather than reactive treatment. Complications from untreated dental decay are one of the most common reasons for potentially preventable acute non‐psychiatric hospital admissions [15, 16]. Resorting to emergency hospital care is expensive for health services. Although targets and strategies exist to tackle health inequalities such as cardiovascular disease and diabetes for people with SMI, there is comparatively less attention on oral health. A dental recovery plan was published in 2024 that shed some attention on this [17]. However, there is an urgent need for action in this area for the proper implementation of this plan. Our team aimed to develop and disseminate a consensus statement highlighting areas of concern and recommending an evidence‐based 5‐year action plan to improve oral health in people with SMI and address oral health inequalities in this area.

## 2 Methods

### 2.1 Consensus Process

The Right to Smile consensus statement was developed by an oral health special interest group, established in 2021 by members of the Closing the Gap Network, a UK Research and Innovation (UKRI)‐funded mental health network, to tackle the health inequalities in people with SMI. A co‐production approach was used to develop the consensus statement where the voices of people with lived experience were central to its development [18]. The oral health special interest group included 20 members (all adults, age > 18 years): five people with lived experience (four male and one female with lived experience of SMI including schizophrenia, bipolar and major depression with psychosis), one male family member/carer of people with lived experience with SMI and 14 experts from different professional disciplines and practice settings. The different professional disciplines and practice settings include two clinical dentists (one male and one female), two consultants in dental public health (both female), one clinical psychologist (male), one consultant psychiatrist (female), one mental health pharmacist (male), one mental health clinical academic (male), one primary care medical doctor (female) and five public health experts and academics (one male and four female). We did not collect any data on socio‐economic condition of people with lived experiences. We did not deliberately exclude anyone, but as this was conducted during the pandemic and the development was done online, it meant that people who did not have access to the Internet were not included. The pertinent statements were developed to stimulate improvement primarily in the United Kingdom, with the hope that this could also be adapted to other countries as evidence shows that oral health inequality in this population exists globally.

**Figure 1:**
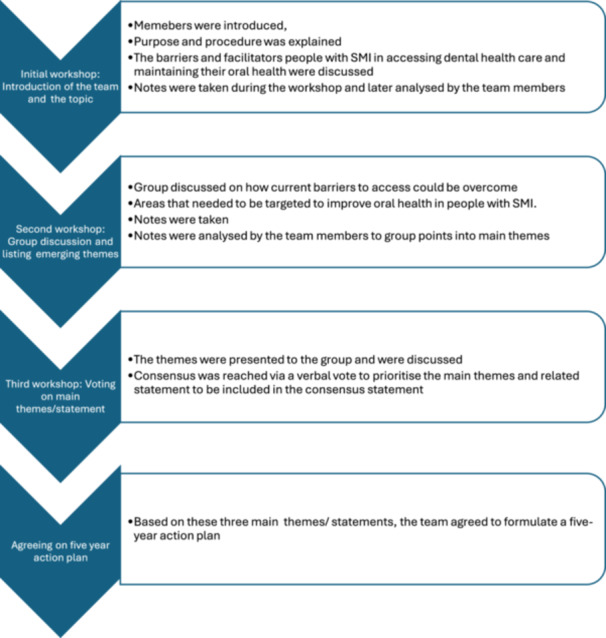
Structure of workshops for developing the consensus statement.

The consensus statement was developed through a series of participatory online workshops held during 2021. These workshops focused on allowing different perspectives and experiences to be heard while being open to criticism, accountability and improvement. We adapted and used the nominal group technique (consensus‐making method) [19]. Figure 1 shows the structure of the workshops used to develop the consensus statement.

The initial workshop (held on 9 April 2021) focused on (i) rapport and relationship building so that group members felt comfortable sharing their views, and (ii) understanding the barriers and facilitators for people with SMI in accessing dental health care and maintaining their oral health. Detailed notes were taken on the points discussed during the workshop. Some of the main discussion points included the following:

- Poor oral health in people with SMI (high rates of tooth loss, dental caries and hospital admissions due to extensive dental treatment).
- Different barriers to accessing dental services (making appointments, stigma issues, attendance hurdles and costs) and maintaining oral health in people with lived experience of SMI.
- Lack of awareness, attitudes and knowledge among dental staff, mental health staff and practitioners from primary care, as well as medical specialties to help and support people with SMI to maintain their oral health.
- Lack of priority in this area in policy, research and training.

**Figure 2:**
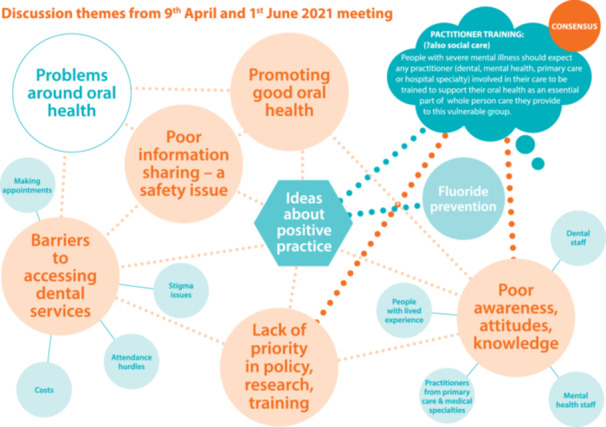
Themes from 9 April and 1 June 2021 meeting.

Building on the emerging themes from the initial workshop, the second workshop (held on 1 June 2021) focused on exploring strategies to address areas of concern, overcoming current barriers to access and identifying specific targets for improving oral health in people with SMI. Detailed notes were taken during the workshop, and the notes were analysed by the team members to group points under the main themes. A visual graphic was prepared to summarise the main themes and their interconnectedness that emerged from the first two workshops (Figure 2).

Building on the emerging themes from the first two workshops, in the final workshop (held on 10 September 2021), the important themes and related statements were presented to the group. There was a set time for discussion around each theme. Consensus was reached via verbal vote to prioritise the themes for inclusion in the consensus statement (i.e., which were the most important to address). The three main themes around health promotion—considering oral health as part of general health and health assessment, improving access to dental services for people with SMI and prioritising oral health in people with SMI in healthcare staff training, policy, practice and research—were selected to be included in the consensus statement. One statement presenting each of the themes was proposed, discussed and agreed upon by the group. Based on these three main themes/statements, the team agreed to formulate a 5‐year action plan. This process of consensus building culminated in the co‐production of a 5‐year action plan based on the three main themes.

### 2.2 The Voice of Those With Lived Experience

The voices of people with lived experience of SMI positively shaped the initiative from its initial conception to the final consensus statement. The statement demonstrates how the voice of lived experience has underpinned the whole initiative, reflected by the choice of title ‘The Right to Smile’ and throughout its content, emphasising the right to a set of service expectations for holistic care. The lived experience voice was central to the process of dissemination, evidenced by active participation through presentations at a launch event and co‐authorship in all subsequent outputs (articles, social media blogs and podcasts).

## 3 Results

The Right to Smile consensus statement [20] first highlighted the right of people with SMI to get help and support for their oral health. This includes the right to equal dental treatment and not to be discriminated against because of their mental health condition; the right to have their oral health valued and supported; the right to consider oral health from the start of their mental health difficulties and treatment; the right to receive information and advice on issues relating to their oral health to make informed decisions; and the right to regular dental check‐ups and dental treatment if they need it.

To achieve these rights, the consensus statement contains three main statements based on three themes (Table 1): consideration of oral health in physical health, improving access to dental services and recognising oral health in healthcare training, systems and structures for people with SMI. Each consensus statement was presented with related recommendations for action. The first statement proposes: ‘Any assessment of physical health in people experiencing severe mental ill health must include consideration of oral health’, as oral health is generally neglected in people with SMI and is often not typically considered as part of physical health check‐ups. The second statement relates to inequity in access: ‘Access to dental services for people with severe mental ill health needs to improve’. Finally, to make a sustainable change in the provision of support to maintain oral health in people with SMI, a structural change is needed in the policy and practice, which leads to the third statement: ‘The importance of oral health for people experiencing severe mental ill health should be recognised in healthcare training, systems, and structures’.

**Table 1:** The consensus statements and related recommendations.

Consensus statements	Agreed recommendations as mentioned in the consensus statement on how this goal can be achieved
‘Any assessment of physical health in people experiencing severe mental ill health must include consideration of oral health’.	» Health promotion advice about healthy eating, tobacco and substance use should initiate discussion and support around oral health. » Carers should be made aware of the importance of oral health for those they support and given advice and information on how they can encourage good oral hygiene. » All annual physical health reviews should include an enquiry about oral health and signposting to a dental service for those not attending regular check‐ups. » As a critical opportunity for prevention, 70% of people will have been signposted to dental care within 6 months of first receiving a diagnosis and medication for severe mental illness.
‘Access to dental services for people with severe mental ill health needs to improve’.	» Dental services must make reasonable adjustments to enable people experiencing severe mental ill health to attend and receive effective oral health care as required by the Equality Act 2010. » At least 60% of people experiencing severe mental ill health should receive regular dental check‐ups consistent with clinical guidelines around dental attendance in the general population. » Dental services should be commissioned to meet the local needs of vulnerable people, which includes those with severe mental ill health, emphasising equity in access to dental services. » People with severe mental ill health should be supported to claim free or subsidised treatment when eligible.
‘The importance of oral health for people experiencing severe mental ill health should be recognised in healthcare training, systems, and structures’.	» All policy, clinical guidelines and training that make recommendations around the physical health of people experiencing severe mental ill health should include oral health. » Dental, medical and other undergraduate teaching curricula should include the understanding and management of oral health problems for people experiencing severe mental ill health. » The joining up of health records across primary and secondary care should include dental records to better support the holistic care of those experiencing severe mental ill health.

## 4 Discussion

The Right to Smile consensus statement sets clear goals to address the previously neglected oral health needs of people with SMI. The consensus statement emphasises the fundamental right of people with SMI to receive adequate and equitable oral health care. This recognition of oral health as a fundamental right is a crucial step towards advocating for improved dental care for people with SMI. The consensus statement focuses on three key themes: (i) consideration of oral health during any assessment of physical health in people with SMI, (ii) improving access to dental services for people with SMI and (iii) recognising the importance of oral health for people with SMI in healthcare training, systems and structures. It recommends a 5‐year action plan based on these three themes.

The first theme relates to the incorporation of oral health assessment into physical health assessments in people with SMI within any healthcare service, as oral health is often not considered in people with SMI. This is likely true of both primary and specialist care [21]. Physical assessments for people with SMI often lack consideration of oral health [22]. The consensus statement reflects a need for a collective effort to recognise and prioritise the importance of oral health within the broader context of mental health care. This recommendation fits well with the target of ensuring annual health checks for 60% of those living with SMI under Core20PLUS5 (a national NHS England [NHSE] approach to inform action to reduce healthcare inequalities at both national and system levels) [23] and NHSE's new guidelines on ‘Improving the physical health of people living with SMI’, where including oral health in the physical health check for people with SMI was recommended [24]. However, along with dental assessment, further tailored self‐care/oral hygiene advice, preventive support and dental care access support are needed for routine clinical dental check‐ups and treatment (as needed) to ensure prevention, early diagnosis and treatment of oral diseases for this population. The consensus statement recognises the need for a system‐level intervention that will help to strategically include oral health in any physical health check in people with SMI and provide further prevention and treatment support as needed.

Healthcare access is a fundamental right for all [25]. However, people with SMI have very limited dental health care access, despite overwhelming evidence of their poor oral health. This demonstrates the inverse care law ‘the availability of good medical or social care tends to vary inversely with the need of the population served’ [26]. The second theme in the consensus statement highlights the need for equitable access to dental care and the need to address the systemic barriers and inequalities that often prevent this population from accessing appropriate dental services. Due to multiple barriers, including the high treatment needs of the SMI population, and the model of NHS dentistry based on high throughput, dental practice–based care appears not to be currently effective for this group of people [11]. Ideally, people with SMI should have access to routine dental visits in line with national guidance [27]. The statement emphasises the need for collaboration and coordination between mental health and dental care providers, which could be crucial to addressing inequalities and ensuring dental access for this population. This should include the commissioning of appropriate services to ensure that the needs of people with SMI are met. Further research should attempt to investigate interventions targeting an effective integration of dental and mental health care to support dental access for this population in UK settings, such as link work interventions [28].

Finally, the third theme reflects a collective effort to recognise and prioritise the importance of oral health within the broader context of training, healthcare systems, research and practice. Evidence suggests that there is a need for training of both mental and dental health care teams in softer communication skills and trauma‐informed practice [29]. Dental professionals need to be able to communicate with people with SMI and understand their difficulties and needs, and the impact that this might have on their oral health. Similarly, mental health professionals should be aware of major dental conditions and side effects, such as dry mouth, that anti‐psychotic and anti‐depressant medications might exacerbate. To address the training needs of mental and dental health professionals, the members of our research team have developed an e‐module titled ‘Psychiatry in Dentistry’, a free interactive resource aimed at undergraduate dental students in use at several UK dental schools. Such training should be available to all dental and mental health professionals. So far, less focus has been placed on oral health among people with SMI in research and practice [30], with multiple barriers to delivery [31]. The consensus statement recognises the need for (i) training of healthcare professionals, (ii) research and evidence‐based practices to guide oral health interventions for people with SMI and (iii) identifying effective strategies, interventions and policies that can improve oral health outcomes in this population.

### 4.1 Strengths and Limitations

The key strength of this consensus statement is that the group drew from a multidisciplinary membership that blended lived experience with a breadth of academic, clinical and policy experience and graphic design skills. We used an iterative process to identify key reflections from this multidisciplinary and diverse group to develop the consensus statement and recommendations. The consensus statement highlights the issue of oral health inequalities in people with SMI and provides guidance for future policy and practice.

The consensus statement was disseminated widely using various platforms. An in‐person launch event of *The Right to Smile consensus statement* was conducted in Manchester in 2022. Following its release in January 2022, The Right to Smile has been endorsed locally, nationally and internationally. Dissemination was further supported through social media (podcasts, blogs, #Right2Smile and #MindYourSmile) and several workshops with NHS practitioners, general dentists and commissioners. So far, *The Right to Smile* has been endorsed by 32 organisations and affiliations locally, nationally and internationally. Some endorsing organisations actively supported dissemination to their membership networks, recognising synergy with their own goals. For example, the Oral Health Foundation provided us with a platform to further disseminate the consensus statement via a podcast between a clinical academic dentist and a person with lived experience to illustrate the impact of oral health on people with SMI and discuss barriers/facilitators to managing their oral health.

Due to limited logistical support and time restraints, we used a pragmatic approach to reach a consensus. We did not use a Delphi process or scoring system, such as a Likert scale to reach an agreement; rather, the consensus was reached via a vote on which three statements from each of the three main themes should be included in the final statement. After achieving agreement on the three main themes, the 5‐year action plan was reached through discussion and deliberation. It was beyond the scope of this study to include all relevant stakeholders, including commissioners and policymakers when developing the consensus statement. The consensus statement was developed during the COVID‐19 pandemic, which meant that utilising established methods was more challenging. Hence, we adopted our pragmatic approach where we held a series of online workshops with breakout rooms for discussion and reached a consensus via online voting.

### 4.2 Recommendations

As most dental diseases are preventable, early diagnosis and treatment could save future extensive treatment. Therefore, the consensus statement promotes both preventive and treatment angles at a system level. Consideration of oral health during any assessment of physical health in people with SMI, facilitation of dental access and recognition of this issue in healthcare training, systems and structure will have a significant role in prevention, early diagnosis and treatment. This will ultimately help in reducing the oral health inequality and inequity in access experienced by people with SMI. The 5‐year action plan outlined in this consensus statement is expected to guide policymakers in making meaningful strides towards improving the oral health and overall well‐being of people with SMI by embracing and implementing the action plan. We acknowledge that some of the recommendations may seem to be ambitious considering the current already stretched dental service in the United Kingdom. However, if the issue continues to be overlooked and if proper action is not taken, the oral health inequality gap in this population will continue to grow, which is unacceptable. The current reformation of the commissioning through Integrated Care Boards (ICB) and plans for the future reformation of NHS dental services have the potential to create an opportunity to think outside the box of current dental service provision for people with SMI. Further research should focus on developing and implementing effective and cost‐effective systems and patient‐level interventions to improve oral health for people with SMI. Policymakers and commissioners should take further action based on the key recommendations of this consensus statement.

### 4.3 Future of the Consensus Statement

The next steps will include the following: (i) capturing any change in the oral health disadvantage suffered by people with SMI and qualitative evidence on the awareness of the consensus statement by policymakers, providers and commissioners; (ii) reviewing and updating the consensus statement in light of emerging evidence; and (iii) linking the statement governance to existing structures to support its impact and sustainability.

## 5 Conclusion

This consensus statement was developed by a group that combines lived experience and professional expertise with a shared commitment to improving the oral health of people with SMI. By taking a rights‐based approach to tackle this neglected but potentially preventable health inequality, the statement provides evidence‐informed recommendations as a call for researchers, services and policymakers to embrace a 5‐year action plan to improve oral health in people with SMI. To quote the consensus statement: ‘Poor oral health should not be an inevitable consequence of experiencing severe mental ill health’.

## Author Contributions

**Masuma Pervin Mishu:** conceptualisation, investigation, funding acquisition, writing–original draft, writing–review and editing, visualisation, methodology, supervision. **Vishal Aggarwal:** conceptualisation, investigation, funding acquisition, methodology, writing–review and editing, supervision. **David Shiers:** conceptualisation, investigation, funding acquisition, methodology, writing–review and editing, supervision, project administration, writing–original draft, visualisation. **Emily Peckham:** conceptualisation, investigation, funding acquisition, writing–review and editing, writing–original draft, methodology, supervision. **Gordon Johnston:** conceptualisation, investigation, funding acquisition, writing–original draft, writing–review and editing, methodology. **Easter Joury:** writing–review and editing. **Carolyn A. Chew‐Graham:** conceptualisation, investigation, funding acquisition, writing–review and editing, methodology, supervision. **Katie Goodall:** project administration, writing–review and editing. **Emma Elliott:** writing–review and editing. **Paul French:** conceptualisation, investigation, funding acquisition, writing–review and editing, supervision, methodology, project administration. **Rebecca Harris:** conceptualisation, investigation, funding acquisition, writing–review and editing. **Louise Laverty:** funding acquisition, writing–review and editing. **Jasper Palmier‐Claus:** conceptualisation, investigation, funding acquisition, writing–original draft, writing–review and editing, methodology, supervision, project administration, visualisation.

## Ethics Statement

Ethical approval was not required as the consensus statement was developed by participatory online workshops.

## Conflicts of Interest

The authors declare no conflicts of interest.

## Data Availability

Data sharing is not applicable to this article as no data sets were generated or analysed during the current study.
